# Substance use among inmates at the Eldoret prison in Western Kenya

**DOI:** 10.1186/1471-244X-13-53

**Published:** 2013-02-13

**Authors:** Daniel WC Kinyanjui, Lukoye Atwoli

**Affiliations:** 1 Department of Mental Health, School of Medicine, Moi University College of Health Sciences, PO Box 4606, Eldoret, 30100, Kenya

## Abstract

**Background:**

Criminal activity and social problems are recognized as important outcomes of substance use and abuse. Little research has been carried out on substance use among prison inmates in Kenya. General population surveys that have examined drug use usually omit this ‘hidden’ population which may offer insight into drug related morbidity and invaluable preventive measures. This study is set out to determine the lifetime prevalence and factors associated with substance use, including the most frequently used substances, among inmates at a government prison in Western Kenya.

**Methods:**

Design: A cross-sectional descriptive study, using the WHO model questionnaire and an additional drug use and effects questionnaire among prisoners at the Eldoret Government of Kenya (GK) prison, Kenya.

Setting: Study was carried out at the Eldoret G.K. prison, with a population of 1325 (1200 males and 125 females) inmates.

Subjects: Three hundred and ninety five prisoners, who gave consent, were selected, consisting of 271 males (68.6%) selected by simple random sampling, and 124 females (31.4%) enrolled consecutively due to their small number. The mean age was 33.3 years (18–72, s.d. 9.8) while the mean number of years of formal education was 8.4 (0–15, s.d. 3.4).

**Results:**

Lifetime prevalence of substance use was 66.1%, while that of alcohol use was 65.1%. Both were significantly associated with male gender, urban residence and higher level of education. The lifetime prevalence of cigarette use was 32.7% while 22.5% admitted to chewing tobacco. Factors significantly associated with tobacco use were male gender, urban residence, being unmarried, younger age, lack of income in the past year. The prevalence of cannabis use was 21%, and this was associated with male gender, urban residence, being unmarried, and being a student in the past year. Other substances used included amphetamines (9.4%), volatile inhalants (9.1%), sedatives (3.8%), tranquillizers (2.3%), cocaine (2.3%), and heroine (1.3%). Users were commonly introduced to the habit by friends (70.8%), immediate family members (13.7%) and other close relatives (6.2%). Among those who reported lifetime substance use the common reasons attributed to the habit were the need to relax (26.5%), relieve stress (24.5%) and confidence to commit a crime (4.5%). Majority of those who reported alcohol use were already suffering ill effects.

**Conclusions:**

There is a high prevalence of substance use among prisoners at the Eldoret G.K. prison. The increased morbidity and unpleasant psychosocial consequences of this habit suggest a need for establishment of substance use management programmes in Kenyan prisons.

## Background

There is increasing evidence for the association between substance use and criminality, including a high prevalence of substance use disorders in prison populations [1-3]. Various studies [4-8] have also demonstrated the need for the enhancement of mental health services to cope with the high number of mentally ill prison inmates, including those with substance related problems. Contemporary studies have further shown that certain personality profiles are linked to some preferential choice of drug [9,10] and that prisons are a high-risk environment for drug initiation [11,12].

There are very few studies on substance use among prisoners in Africa as compared to their western counterparts. This is despite literature repeatedly showing criminal activity and social disorder as major outcomes of substance use [3,13,14]. One of the very few studies on this subject in Africa found that the lifetime drug use among prisoners in Uganda was 65% and that the most commonly abused drugs were tobacco/cigarettes (90%), marijuana (49%), khat/mairungi i.e. *catha edulis* ( 17%) and alcohol (2%). Unlike earlier studies from the higher income countries, there was no reported injecting drug use (I.D.U.) in Uganda [15].

Very little similar work has been carried out in Kenya, or even in the wider Eastern Africa region. The importance of carrying out local studies cannot be overemphasized, since each region probably has unique socio-demographic, crime and substance use profiles.

General population surveys [16-19] in Kenya have found relatively high lifetime alcohol use rates as compared to other substances, with especially low rates of injecting drug use. However, a study [20] commissioned by the United Nations Office on Drugs and Crime and conducted by the University of Nairobi revealed that over and beyond the findings in earlier studies, heroin, cocaine and amphetamines were also abused, with injecting drug use high in the capital city Nairobi and coastal towns of Malindi and Mombasa.

Whereas a general population survey will provide useful data on trends of substance use in a country, it would omit the ‘hidden’ prison population which may offer a lot more insight into drug related morbidity and invaluable preventive measures. This study set out to fill the knowledge gap by determining the prevalence of substance use as well as the associated factors and outcomes among prisoners at the Eldoret G.K. prison in Uasin Gishu County, Kenya.

## Methods

### Site

The study was carried out at the Eldoret G.K. prison, situated 1.5 KM off Iten road, northeast of the central business district in Eldoret town. Eldoret town is 320 KM northwest of the capital city of Kenya, Nairobi. The prison was established in 1963 with an intended capacity of 600 inmates. At the time of the study, however, the prison had an inmate population of about 1325 (1200 males and 125 females) and 600 staff members.

### Participants

Three hundred and ninety five prisoners were selected, 271 being male. Due to the large number of male compared to female prisoners, all female prisoners were offered the opportunity to participate in the study in order to allow for comparison with the men. A total of 124 female prisoners accepted to participate, signed the consent and were recruited for the study. Only one female declined to participate.

### Sampling procedure

A sample of 383 was calculated using Fisher’s formula (a confidence interval of 95% and the prevalence rate of lifetime drug use assumed at 50% due to paucity of local studies). Desired sample size was calculated for a population less than 10,000, resulting in a minimum sample size of 291 male and 92 female inmates, giving a minimum sample size of 383. For reasons given above, we chose to include all female inmates and randomly sampled the male inmates.

A serialized list of male prisoners was obtained and every 4^th^ male prisoner was thereafter selected for the study, until the desired sample size of 291 male prisoners was reached. All male prisoners selected to participate in the study accepted and signed consent.

### Design

A cross-sectional descriptive survey design was used involving the administration of two instruments.

The World Health Organization interviewer administered Model Core questionnaire was used to gather information on the lifetime and current use of various drugs and an additional questionnaire based on introduction to drug use, reasons best explaining drug use, and problems attributed to drug use. The WHO core model questionnaire was developed through a cross-cultural field assessment at six sites (one in each of the six WHO regions), i.e. Egypt, Greece, India, Mexico, Malaysia and Zimbabwe [21].

The additional questionnaire was used to gather data on other aspects of drug use. It contained three questions:

1. Who introduced you to drug use? Possible responses for this question were: a) A friend; b) A parent; c) A sibling; d) Other relative; e) Other (specify).

2. Which reason(s) explain why you use the substance you use? Possible responses for this question were: a) To relax; b) To relieve stress; c) To be accepted by peers (peer pressure); d) Excess pocket money; e) Desire to experiment; f) They are easily available; g) To feel normal; h) For confidence to commit offence; i) Others (Specify).

3. What problems have you had that you attribute to your use of the substance(s)? Possible responses for this question were: a) Quarrel or argument; b) Scuffle or fight; c) Accident or injury; d) Loss of money or other valuable items; e) Damage to objects or clothing; f) Problems in your relationships with your parents; g) Problems in your relationship with your friends; h) Problems with your spouse; i) Problems in your relationship with teachers; j) Performed poorly at school or work; k) Victimized by robbery or theft; l) Trouble with police; m) Hospitalised or admitted to an emergency room; n) Engaged in sex you regretted the next day; o) Engaged in unprotected sex; p) Blackouts or flashbacks; q) Medical problems e.g. memory loss, hepatitis, head injury, bleeding etc.; r) Other (specify).

Both the WHO model core questionnaire and the additional questionnaire have been used in Kenya to collect data on substance use [22,23]. However, an additional option, “confidence to commit an offence”, was added to the reasons for using a substance in order to explore this possibility among the prison inmates. Lifetime use in this study refers to having used the substance at any point in the respondent’s life.

### Procedure

The study was carried out between June and August 2011. The Institutional Research and Ethics Committee of Moi Teaching and Referral Hospital and Moi University conducted ethical review of the study. Approval was also sought and granted from the Commissioner of Prisons, Ministry of Home Affairs in the Vice Presidents Office in Kenya with subsequent training of a research assistant on the administration of the questionnaires, prior to onset of the study. All selected to participate in the study were then grouped and briefly addressed concerning the purpose and methodology with assurance of confidentiality concerning the information collected. The individual interviews were then conducted in an environment which accorded safety and auditory privacy to enable participants to talk openly about their views.

### Data storage and analysis

Collected data underwent cleaning and storage in a Microsoft Excel database and subsequently analyzed using SPSS version 17.0. Descriptive statistics were used to compute means and standard deviations for numerical variables as well as frequencies for nominal and ordinal variables. Significance of association between various variables and substance use was tested using the Chi square test statistic (x^2^) and a finding of p< 0.05 was considered statistically significant. The variables strength of association with substance use was also ascertained through multiple logistic regression analyses.

## Results

### Socio-demographic distribution

Three hundred and ninety five prisoners participated in the study, 68.6% were male. The mean age was 33.3 years (18–72 yrs, s.d. 9.8) and the mean number of years of formal education was 8.39 years (0–15 yrs, s.d. 3.4). As shown in Table 1, majority had not been students and most of them had a source of income, over the last one year.

**Table 1:** Socio-demographic distribution

**Variable**	**N (%)**
**Gender**	
Male	271 (68.6)
Female	124 (31.4)
**Residence**	
Rural area	183 (46.3)
Urban area	212 (53.7)
**Marital status**	
Married	234 (59.2)
Unmarried	161 (40.8)
**Employment status**	
Unemployed	184 (46.6)
Employed	211 (53.4)
**Student**	
Yes	42 (10.6)
No	353 (89.4)
**Income**	
No	106 (26.8)
Yes	289 (73.2)

### Prevalence of substance use

The prevalence of lifetime substance use among prisoners at the Eldoret G.K. Prison was 66.1%. There was no statistically significant difference in age between those reporting lifetime substance use and those not reporting it (33.0 years vs 33.8 years, t=0.771, p= 0.441).

Those reporting lifetime substance use had a statistically significantly higher level of education (8.7 years) than those not reporting it (7.9 years), t=2.159, p=0.031. As shown in Table 2, the other variables that were significantly associated with lifetime substance use included male gender ( p< 0.001) and living in an urban residence ( p< 0.001).

**Table 2:** Summary of the distribution of lifetime substance use and most frequently used substances

	**Lifetime substance use**			**Alcohol use**			**Cigarette use**			**Tobacco chewing**			**Cannabis use**		
**Variables (N)**	**Prevalence** **(%)**	**X**^**2**^	**P value**	**Prevalence (%)**	**X**^**2**^	**P value**	**Prevalence ( %)**	**X**^**2**^	**P value**	**Prevalence (%)**	**X**^**2**^	**P value**	**Prevalence (%)**	**X**^**2**^	**P value**
**Gender**															
Male (271)	72.3	15.038	<0.001*	70.8	12.711	<0.001*	37.6	9.735	0.002*	25.5	4.245	0.039*	23.6	3.526	0.060
Female (124)	52.4			52.4			21.8			16.1			15.3		
**Residence**															
Rural area (183)	54.1	21.822	<0.001*	54.1	18.035	<0.001*	20.8	21.93	<0.001*	14.2	13.535	<0.001*	12.0	16.606	<0.001*
Urban (212)	76.4			74.5			42.9			29.7			28.8		
**Marital status**															
Married (234)	63.7	1.476	0.224	62.4	1.801	0.180	25.6	12.854	<0.001*	16.2	13.022	<0.001*	15.8	9.356	0.002*
Unmarried (161)	69.6			68.9			42.9			31.7			28.6		
**Employed**															
Yes (211)	70.1	3.341	0.068	69.2	3.401	0.065	29.9	1.615	0.204	19.0	3.315	0.069	18.5	1.746	0.186
No (184)	61.4			60.3			35.9			26.6			23.9		
**Student**															
Yes (42)	73.8	1.254	0.263	73.8	1.582	0.209	52.4	8.312	0.004*	33.3	3.142	0.076	47.6	20.045	<0.001*
No (353)	65.2			64.0			30.3			21.2			17.8		
**Income**															
Yes (289)	66.1	0.000	0.992	65.4	0.053	0.818	29.8	4.119	0.042*	17.6	14.721	<0.001*	17.6	7.35	0.007*
No (106)	66.0			64.2			40.6			35.8			30.2		

*Statistically significant (p< 0.05).

### Specific substance use

Among the 395 respondents who answered this question, 65.1% reported lifetime alcohol use with a mean age at first drink of 22.6 years (7–56, s.d. 6.3). Those reporting lifetime alcohol use showed a statistically significantly higher level of education (8.7 years) than those who did not (7.9 years), t= 2.192, p=0.029. As shown in Table 2, male gender (p< 0.001) and living in an urban residence (p< 0.001) were also significantly associated with a lifetime alcohol use. A total of 10.4% of respondents reported current alcohol use (had an alcoholic drink in the preceding week).

The most commonly reported effects attributed to alcohol use included: quarrelling/ arguing (57.0%), trouble with the police (55.9%) and scuffle or fights (47.6%). Table 3 shows the reported effects attributed to alcohol use.

**Table 3:** Effects of alcohol use

**Effect**	**Percentage**
Quarrel or argument	57.0%
Trouble with police	55.9%
Scuffle or fight	47.6%
Blackout or flashback	44.3%
Loss of money/valuable items	43.8%
Accident or injury	40.8%
Discord relationship with spouse	35.2%
Discord relationship with parents	31.6%
Poor performance at work	30.1%
Unprotected sex	26.1%
Engaged in sex you regretted the next day	24.6%
Damage to property	22.8%
Discord relationship with friends	21.0%
Medical problems	17.5%
Victimized by robbery or theft	11.6%
Discord relationship with employer	10.4%
Hospitalized/ admitted as an emergency	10.4%
Discord relationship with your teachers	6.1%

The lifetime prevalence of cigarette use was 32.7%, and 14.0% of the respondents were considered current smokers (in the past week). The mean age at first cigarette was 21.2 years (12–35, s.d. 5.0) with the mean age of those reporting lifetime smoking (31.3 years) being statistically significantly lower than non-smokers (34.3 years), t=2.879, p=0.004. Males had a significantly higher lifetime cigarette smoking rate than the females (p=0.002).

As illustrated in Table 2, those who were not married (p<0.001), lived in an urban residence (p<0.001), had no source of income (p=0.042), and were students (p=0.004) over the past year showed statistically significantly higher rates of cigarette use than their counterparts. The lifetime prevalence of chewing tobacco was 22.5% with the mean age at first chewed tobacco as 24.3 years (14–45, s.d. 6.0). Those reporting current tobacco chewing (in the past week) were 7% of the respondents.

Mean age of lifetime tobacco chewers was significantly lower than that of non chewers (31.2 vs 33.9 years, t=2.331, p=0.02) and males had a statistically significantly higher prevalence of chewing tobacco than the females (p=0.039). Those who were unmarried (p<0.001), had no source of income (p<0.001), were living in an urban residence over the past year (p<0.001) also had statistically significantly higher chewing rates. Table 2 also shows the relationship between tobacco chewing and various variables.

The lifetime prevalence of cannabis use in this study was 21%. The regular cannabis use (in the past month) prevalence was 5%, while the age at first cannabis use was 21.7 years (range 9–46, s.d. 5.7). Those living in an urban residence (p<0.001), were unmarried (p=0.002), students (p<0.001) and with no source of income (p=0.007) over the past year showed statistically significantly higher lifetime cannabis use rates than their counterparts. Level of education (t=0.877, p=0.381) and age (t=1.641, p=0.102) of the respondents showed no statistically significant association with lifetime cannabis use. There was no statistically significant association between lifetime prevalence of cannabis use and any of the other variables. The relationship between cannabis use and these variables has also been illustrated in Table 2.

Further analysis of the data was carried out to determine the strength of association between various variables and substance use. Table 4 below shows results for the multiple logistic regression models.

**Table 4:** Summary of the logistic regression analyses

**Variables**	**Lifetime substance use**			**Alcohol use**			**Cigarette use**			**Tobacco chewing**			**Cannabis use**		
	**Odds ratio**	**[95% CI]**		**Odds ratio**	**[95% CI]**		**Odds ratio**	**[95% CI]**		**Odds ratio**	**[95% CI]**		**Odds ratio**	**[95% CI]**	
**Female vs Male**	0.39*	0.24	0.64	0.41*	0.25	0.67	0.32*	0.18	0.56	0.44*	0.24	0.80	0.49*	0.26	0.92
**Urban vs Rural**	2.84*	1.80	4.49	2.45*	1.56	3.84	2.85*	1.75	4.63	2.43*	1.41	4.18	2.52*	1.43	4.45
**Unmarried vs Married**	1.40	0.86	2.28	1.67*	1.03	2.71	2.43*	1.47	4.01	2.21*	1.28	3.81	2.02*	1.15	3.55
**Employed vs Unemployed**	1.17	0.69	1.98	1.32	0.79	2.23	0.72	0.42	1.27	0.89	0.47	1.67	0.87	0.46	1.67
**Student vs Not student**	1.03	0.47	2.25	1.00	0.47	2.14	1.63	0.82	3.24	1.27	0.61	2.67	3.05*	1.51	6.14
**Income vs No income**	0.90	0.50	1.63	1.05	0.59	1.89	0.99	0.54	1.79	0.52*	0.27	0.97	0.66	0.34	1.28

*Statistically significant (p< 0.05).

We observe that controlling for other factors gender; residence and marital status were statistically significantly associated with the various outcomes except for marital status which was not associated with overall substance use. Specifically, females had a lower odds of using any substance compared to males OR= 0.39, 95% CI [0.24, 0.63] holding other factors constant. All else being equal those living in urban areas had a higher odds of using any substance compared to those living in rural areas OR= 2.84, 95% CI [1.80, 4.49]. While holding other factors constant unmarried individuals were more likely to use alcohol, cigarettes, chew tobacco and use cannabis compared to those that were currently married. Student status was only found to be significantly associated with cannabis with student having higher odds of using cannabis compared to non students OR= 3.048, 95% CI [1.513, 6.142] holding other factors constant. While controlling for other factors, income status was only found to be significantly associated with tobacco chewing with those with some source of income having lower odds of chewing tobacco compared to those with no income OR= 0.516, 95% CI [0.273, 0.974].

Other substances reported included amphetamines (*catha edulis*) 9.4%, volatile inhalants (*glue*) 9.1%, sedatives 3.8%, tranquillizers (2.3%), cocaine (2.3%) and heroine (1.3%).

### Introduction to substance use

Among those who admitted to lifetime substance use, 70.8% had been introduced to substance use by friends, 6.2% by close relatives, and 13.7% by members of the nuclear family (sibling 9.2%, parent 4.5%). A few of the respondents (9.3%) did not disclose this information.

### Reason for substance use

Among those who admitted to lifetime substance use, the common reasons attributed to the habit included relaxation (26.5%), to relieve stress (24.5%), acceptance by peers (14.9%), experimentation (13.4%), availability (8.0%), to feel normal (5.2%) and for the confidence to commit crime (4.5%). A few of the respondents (3%) declined to offer this information.

## Discussion

The lifetime prevalence of substance use in this study was 66.1%, a rate that is comparable to the 65% reported in the study among prisoners in Uganda [15]. In a setting such as this where prison mental health services are grossly inadequate, the high prevalence of substance use is worrying given the well-established relationship between substance use, mental disorders and even personality disorders [6,7,24-26]. This gap is however not unique to Kenya or other low income countries, as studies from higher income countries have also demonstrated the need for an increase in the provision of mental health services to cope with the high number of mentally ill inmates [4,5,8].

Most substance users in this population were introduced to the habit by friends and family members. Other studies in the region have reported similar findings [22,27]. The implication of this finding is that the approach to drug awareness campaigns should incorporate not only the youth but their parents and even older relatives in the same forum.

Those with a higher level of education, had lived in urban settings and were of the male gender had significantly higher rates of lifetime substance use, suggesting that schools and urban environments provide more opportunities for initiation into substance use for many young men. Oteyo and Kariuki in a 2009 study carried out in nine secondary schools in this same region found that among other factors peer group and family influence had the greatest contribution to high alcohol and cigarette use [27]. However, the association between marital status and lifetime substance use in the current study was confounded by the other variables.

The lifetime prevalence of alcohol use in this study was 65.1%. This is much higher than that reported in the earlier Ugandan Prisons study [15]. The possibility of some access to alcoholic beverages in the Kenyan prison setting cannot be completely discounted in an environment where ‘special operations’ by prison authorities regularly find many illicit belongings in the prison cells, including mobile phones, coins, cannabis and other drugs [28]. It is however comparable to the prevalence of 62% reported by Othieno et al. in a study of outpatients attending several Nairobi primary health care facilities [18].

Surprisingly, a current alcohol use was reported by some of the participants in this study. Due to the restriction of access to alcohol in a prison environment, this finding probably reflects the fact that some inmates had stayed in the prison for short enough durations of time to have had a drink before incarceration. However, the suggestion of illicit acquisition of alcohol cannot be discounted [28].

Notable though was the earliest reported age at first drink of 7 years. This is even younger than what has been reported in earlier studies in this region [19,22]. The fact that most alcohol consumed in Kenya comes from small-scale illicit brews that are produced and sold in homes may explain the early availability of alcohol to children in such homes [29].

Some of the negative effects attributed to alcohol use in this study included engaging in quarrels and arguments, scuffles and fights, unprotected sex, property damage, trouble with the police, suffering blackouts, medical problems and discord relationships. Reported outcomes of early onset alcohol use include the possibility of dependence, other mental illnesses and difficulties adjusting in later life [24,30,31]. In the present study, alcohol use was also associated with higher levels of education, being unmarried and living in an urban environment, further reflecting the role of peer influences and easy availability of alcoholic beverages to young people.

The habit of chewing tobacco and cigarette smoking was also reported by some of the participants in this study. The type of tobacco commonly chewed was ‘kuber’, a brand name for various preparations that may contain tobacco, slaked lime, areca nuts and betel quid leaf [32]. Some participants also reported current tobacco chewing and smoking, suggesting that the illicit acquisition of these products cannot be discounted [28], although the finding may be as a result of shorter durations of incarceration.

Cigarette use was significantly associated with male gender, younger age, urban residence and being unmarried. Student and income status association with cigarette use were shown to be confounded by other variables. Being married is a modifiable variable and seems to be a protective factor in relation to the habit. Chewing tobacco might also be a cheaper option for those younger unmarried males in an urban residence with the need to experiment. As it may not be fashionable to be seen chewing tobacco among those with a source of income, smoking cigarettes was more acceptable in this population. Studies have suggested that cigarette use appears fashionable, and is often the first drug many young people are exposed to, before diversifying to other substances [16,22].

The lifetime prevalence of cannabis use was 21%, a finding similar to the rate of 26.7% found in a 2003 study in France [33], but much lower than that (49%) in the Ugandan study [15]. Similarly, Odek Ogunde et al. [34] reported a rate of 19.7% in a study among students at a Kenyan university. Factors associated with lifetime cannabis use included being a student, unmarried, living in an urban residence and of the male gender. The implication is that peer influence and availability and accessibility of cannabis play a role in cannabis initiation and use. Further research is necessary to examine the exact relationship between these variables and cannabis use.

Some participants in the current study reported cannabis initiation as early as at the age of 9 years. Considering the evidence of psychological dependence and high risk of mental illness associated with cannabis use [25,26], this finding needs to be taken into consideration in the formulation of mental health promotion campaigns.

Despite the relatively low lifetime prevalence for the use of other substances reported in this study, the findings are much higher than those determined among high school, college and university students in the same region [22,34]. However, this is in keeping with findings in correctional facilities elsewhere, further supporting the association between substance use and criminality [1,3,13]. Additionally, cocaine and heroin use was also reported in the current study unlike in the earlier Ugandan study [15]. This is a worrying trend that requires further assessment which should specifically include examining for the documented complications of injecting drug use [20].

Reasons given for using substances include to relax, to relieve stress, to experiment, to feel normal, to be accepted by peers, easy availability of the substances and the confidence to commit a crime. These reasons are similar to those given by substance users in other studies, except for the additional ‘confidence to commit a crime’ [22,35,36].

The results from the present study suggest a need for the enforcement of measures aimed at reducing availability and accessibility of substances, given that it has been suggested before that the substance use problem in many countries is largely due to a failure of the enforcement of existing regulations [29].

Among the limitations in this study was the fact that the period of incarceration for the prisoners was not determined. This may have resulted in the reports of current substance use, especially among the new arrivals in the prison. It also makes it difficult to arrive at any conclusions concerning the availability of alcohol and other substances in the prison environment.

Due to the cross-sectional nature of this study, it is not possible to make any conclusions as to the causal relationships between the various variables and substance use. A longitudinal study would be better suited for this sort of analysis.

There are two categories of prisons in Kenya; the first is a general prison, such as the one at which this study was carried out, while the second is the maximum security category. The crime profiles are different at these two prison types, and it therefore follows that our findings cannot be generalised to the entire prison population in the country. However, this study provides valuable insights that may serve as the basis for the conduct of a nationwide study of substance use among inmates in Kenyan prisons.

Information concerning the type of crimes that had been committed by the clients prior to incarceration was also not collected in this study. This would have been invaluable evidence- based data in determining any association between drug crimes or related drug use and incarceration. Further, it would have enabled comparison with findings from other settings.

Finally, in this study, there was no attempt to screen for or diagnose mental disorders. The complex relationship between mental illness and substance use suggests that any future study of this nature must take into consideration the need to screen for mental disorders as well. This would provide useful planning information as regards financial and human resource allocation for mental health services in Kenyan prisons.

## Conclusions

This study has demonstrated a high prevalence of substance use among inmates in a Kenyan prison. Alcohol, cigarettes and cannabis are the most commonly used substances with injecting drug use also being reported. Some of the negative effects attributed to alcohol use in this study included scuffles and fights, unprotected sex, suffering blackouts, medical problems and discord relationships. It is recommended that services geared towards education and management of substance use among prisoners in Kenya be instituted and early intervention through involvement of the family and significant others would be useful in reducing the burden of substance use in the country.

## Competing interests

The authors declare that they have no competing interests.

## Authors’ contributions

Both authors were involved in the conceptualization of the study idea, development of the study design and instruments, supervision of data collection, analysis and preparation of the final manuscript. Both authors read and approved the final manuscript.

## Pre-publication history

The pre-publication history for this paper can be accessed here:

http://www.biomedcentral.com/1471-244X/13/53/prepub
